# Influence of Interaction between Microcracks and Macrocracks on Crack Propagation of Asphalt Concrete

**DOI:** 10.3390/ma17122877

**Published:** 2024-06-12

**Authors:** Jianhuan Du, Jingang Wang, Zhu Fu

**Affiliations:** School of Architecture and Civil Engineering, Chengdu University, Chengluo Avenue No. 2025, Chengdu 610106, China; wangjingang@cdu.edu.cn (J.W.); fuzhu@stu.edu.cn (Z.F.)

**Keywords:** road engineering, asphalt concrete, microcracks, crack density, interaction effect, crack tip stress field

## Abstract

This paper aims to reveal the interaction relationship between microcracks and macrocracks and the influence of the interaction on the crack propagation behavior. A theoretical model of asphalt concrete was established for the interaction between microcracks with different crack densities and a macrocrack. And a meso-structure model of AC-13 dense-graded asphalt concrete was established by combining the Talyor medium method and the DEM (discrete element method). Macro and micro parameters, such as the stress–strain characteristics, crack evolution parameters, and crack tip stress field, were obtained through a semi-circular bend virtual test and used to study the characteristics of crack propagation under the interaction between microcracks and the macrocrack. The results indicate that the interaction has an effect throughout the process of asphalt concrete damage, and shows shielding and acceleration effects as the microcrack density changes. When the microcrack density is low (*f*_3_ ≤ 0.8), the crack propagation process, which is affected by the interaction effect, exhibits significant differences, and the interaction effect shows the shielding effect. When the microcrack density is high (*f*_3_ > 0.8), the fracture stage is mainly affected by the interaction effect, which shows the acceleration effect. The results provide a predictive theoretical and numerical model for low-temperature cracking of asphalt pavement, and theoretical support for the design, maintenance, and upkeep of long-life pavement.

## 1. Introduction

Asphalt concrete is a non-homogeneous composite material with a specific spatial structure. It is a mixture of asphalt mortar, aggregates, and voids. Traditional laboratory tests were carried out to reveal the relationship between fatigue cracking of the asphalt concrete and various factors, such as different additives [1], fiber content [2], and reclaimed asphalt pavement (RAP) content [3]. Moreover, combined with ENDB (Edge Notched Disc Bend) and SEM (Scanning Electron Microscopy), it revealed the effect of asphalt-to-cement ratio (A/C) on the stress intensity factor of materials [4]. Moreover, aiming at warm mix asphalt (WMA), the viscoelastoplastic continuum damage (VEPCD) model was established to illustrate the cracking sensitivity of asphalt concrete [5]. And the four-point bending test [6] was carried out to analyze the effect of mineral additives on the asphalt concrete viscoelastic behavior from the density, gradation, and strength properties, respectively.

However, because of the microcrack randomness in the spatial distribution of asphalt concrete, the stress field is different from the stress field of asphalt concrete with a single crack, ultimately leading to changes in the fracture behavior of asphalt concrete [7,8]. The Muskhelishvili complex function method [9,10], the small parameter method [11,12,13], the distribution dislocation method [14], the averaging method [15], and the crack line method [16] have been used to obtain the analytical solution of the stress intensity factor of co-linear double cracks and cyclic co-linear multi-cracks in an infinitely large flat plate under uniform load and concentrated load. These methods have also been used to determine the connection between the interaction effect and crack propagation from the perspective of the special distribution of microcracks. However, in practical engineering, the randomness of the spatial distribution of microcracks hinders the calculation of the analytical solution of the stress intensity factor.

Therefore, in studying the impact of penetrating crack clusters and surface crack clusters on macrocrack extension evolution, cracks have been mainly assumed to be in-plane cracks or surface cracks under plane stress or plane strain conditions. Jiang [17,18] and Renshaw [19] used the projection method to establish a wing-like double-crack model by consolidating the penetrating crack group into a single crack. The model was used to investigate the effect of double-crack interactions and the interaction mechanism between secondary crack populations and macrocracks, considering parallel equal-length edge cracks and parallel unequal-length centering cracks, respectively. Moreover, the wing-like double-crack model has been applied to parallel-biased double cracks [20], co-linear or biased double cracks [21], short cracks [22], and triple co-linear cracks [23], respectively, where the interaction mechanism between penetrating crack groups and main cracks was determined by analyzing the influence of penetrating crack groups on the main crack propagation. Additionally, Kamaya [24] merged surface crack clusters into a single crack using the envelope method; the author considered the relative position of the double cracks to reveal the shielding effect of parallel surface-biased double cracks on the macrocrack propagation. Moussa [25] considered the size of double cracks to analyze the influence of the load type on the shielding effect of the parallel surface-biased double cracks. Meanwhile, from the perspective of secondary surface cracks, studies have revealed the influence of the depth of the secondary surface crack on the interaction effect between the secondary surface crack and the deflected primary crack [26,27,28].

The Finite Element Method (FEM) and lab tests have also been combined to study the shielding effect of coplanar double cracks on macrocrack extension, considering various factors, such as microcrack spacing [29,30], the relative spacing between cracks [21,31], crack size [32], and crack depth [33]. The local fracture energy index was utilized to characterize the transient impact of a specific number of randomly distributed microcracks on macrocrack propagation through the integration of Digital Image Technology (DIT) and Acoustic Emission (AE) [34]. The FEM was used to introduce microcracks with a consistent orientation in the constant and crack extension regions to establish the correlation between macroscopic crack extension and toughening mechanisms from the perspective of the number of microcracks [35]. Furthermore, from the perspective of the toughening modulus of fiber-reinforced composites, the toughening effect of different microcracks with a unidirectional distribution was determined by analyzing the effect of unidirectionally distributed microcracks on the macrocrack propagation considering different fiber spatial orientations [36].

In summary, most studies have focused on revealing the effect of the interactions between the penetrating double crack, the surface (buried) double crack, and the main crack. These studies have primarily used the projection method or envelope method, which combines a group of microcracks into a single crack. Meanwhile, a combination of lab experiments and the FEM has been used to describe the toughening effect of microcracks in terms of microcrack amount and orientation. However, changes in the stress field at the macrocrack tip resulting from the microcracks’ randomness in the spatial distribution of asphalt concrete influence the evolutionary behavior of the macrocrack’s extension.

Therefore, according to previous research results, the AC-13 suspended dense structural asphalt concrete at −20 °C was taken as the research object in this study. The Talyor medium method and the discrete element method (DEM) were combined to establish a meso-structural model of asphalt concrete with different microcrack densities; crack density was introduced to characterize the spatial distribution of the model’s internal microcracks. Additionally, the virtual semi-circular bend (SCB) was used to analyze the influence of different crack densities on the propagation behavior of the macrocrack considering macro and micro parameters, such as stress–strain curve, effective modulus change, and crack zone stress field. Moreover, we established the relationship between the distribution characteristics of microcracks and the interaction effect between cracks.

## 2. Effective Modulus of Asphalt Concrete

### 2.1. Influence of Morphological Characteristics of Macrocracks

**Figure 1:**
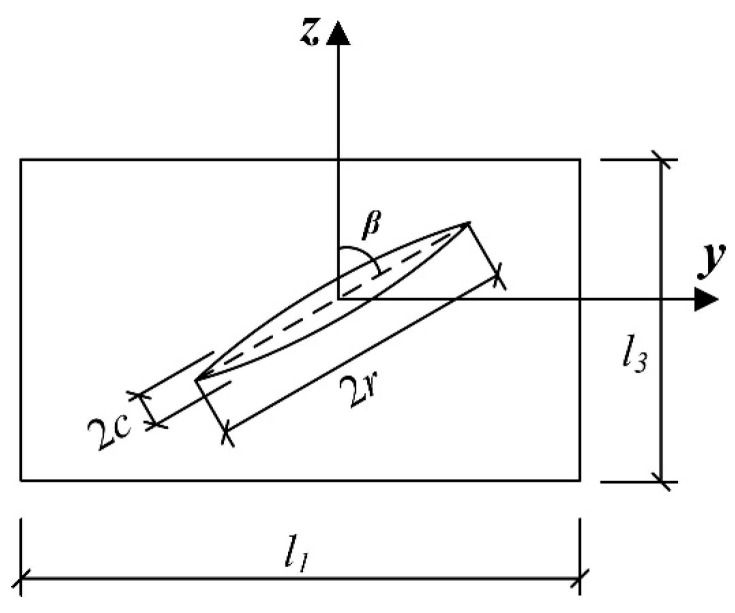
Schematic of a thin elliptical crack in the RVE unit.

Under the action of loading, changes in the macrocrack configuration of asphalt concrete result in corresponding changes in the free energy of the material, which alters the fracture form of the asphalt concrete. The macrocrack in the asphalt concrete is taken as a representative volume element (RVE), assuming that the macrocrack has an initial deflection angle *β*, a short axis size *c*, and a long axis size *r* in the other two directions (as shown in Figure 1). Thus, the geometrical properties of the crack can be defined by Equation (1).

Based on the Tanaka–Mori theory [37], the effective modulus *E_T_* of asphalt concrete containing a thin elliptical crack can be expressed as follows:

(1) $$\frac{E_{T}}{E}=\frac{1}{1+\frac{f_{1}}{f_{2}}(\frac{4(1-v)r}{\pi c})}$$

where *f_1_* and *f_2_* denote the volume fractions of the main crack and asphalt concrete, respectively, and *f*_1_ *=* 1 − *f*_2_; ν denotes the Poisson’s ratio of the material; *E* denotes the modulus of the undamaged asphalt concrete; and *E_T_* denotes the effective modulus of the asphalt concrete containing the main crack.

The geometric parameters of thin elliptical cracks can also be expressed as the ratio between the volume fraction *f*_1_ and the crack shape ratio *α*:

(2) f1α=4πr2c3l12l3·rc=43πp32tanβ

where *p* = *πr*^2^/$l_{1}^{2}$ represents the ratio of the crack area to the bottom area of the representative volume unit RVE; tan*β* = *l*_1_/*l*_3_ represents the length–height ratio of the representative volume unit RVE, which is related to the initial deflection angle of the crack *β* (the angle between the direction of the main crack and the direction of the load loading), 0° ≤ *β* ≤ 90°; α denotes the shape ratio of the thin ellipsoidal crack; and *α* = *c/r*.

The following ratio can be obtained by combining Equations (1) and (2):

(3) ETE=11+16(1−v)tanβ3f2(pπ)32

**Figure 2:**
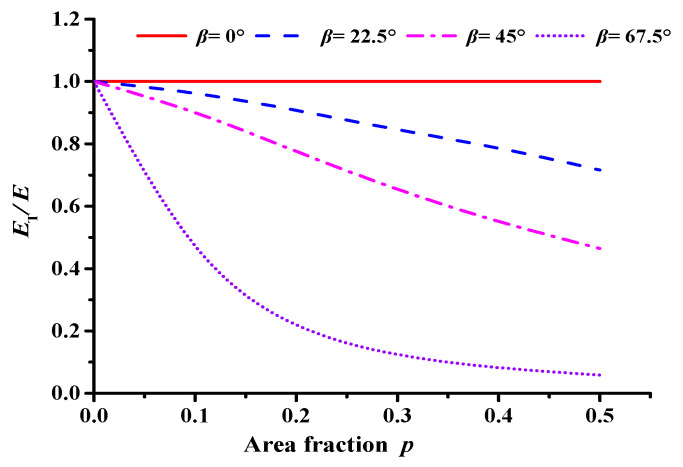
The relationship between effective modulus and parameters *p*, *β*.

From Equation (3), for asphalt concrete with thin elliptical cracks, the effective modulus *E_T_* is related to the crack length *r* and the distribution state of the cracks in space. The Poisson’s ratio *v* of asphalt concrete generally ranges between 0.25 and 0.45; and the majority of the existing studies assumed a constant Poisson’s ratio value of 0.35 [38,39]. Besides considering the Poisson’s ratio of asphalt concrete decreases in the low-temperature condition, the Poisson’s ratio *v* = 0.3 is taken as an example. Meanwhile, for asphalt concrete containing only a single macrocrack, the volume fraction of its macrocrack is negligible, i.e., *f*_2_ = 1. Equation (3) shows the relationship between the area fraction *p*, crack deflection angle *β*, and effective modulus *E_T_* of asphalt concrete, which is illustrated in Figure 2.

Figure 2 shows that at a fixed initial deflection angle *β*, the effective modulus of asphalt concrete decreases slowly and nonlinearly as the area fraction *p* increases gradually. Additionally, at a fixed crack length *r*, the effective modulus of asphalt concrete decreases significantly as the initial deflection angle *β* decreases. The figure also shows that in terms of the geometric size of the main crack, its initial deflection angle *β* greatly affects the effective modulus of the asphalt mixture.

### 2.2. Influence of Interaction Effect between Microcracks and the Macrocrack

The following assumptions are made regarding microcracks at the tip of the macrocrack in asphalt concrete [40]:

1. Each microcrack is uniformly open, without considering microcrack closure and crack surface friction;
2. The microcrack orientation is random and no dominant microcrack orientation exists;
3. Only weak interactions exist between neighboring microcracks.

Accordingly, by combining Equation (3) with the Talyor medium method [41], a connection can be established between the effective modulus of asphalt concrete and the interaction effect between microcracks and the macrocrack:

(4) $$\frac{E^{\prime }}{E_{T}}={\left[1+f_{3}\frac{16(1-v^{2})(10-3v)}{45(2-v)}\frac{E}{E_{T}}\right]}^{-1}$$

where *E*′ denotes the effective modulus of asphalt concrete containing microcracks and the macrocrack; and *f*_3_ denotes the crack density parameter of the microcracks, which is related to the number of cracks, crack orientation, and crack geometry parameters.

Based on Equation (4), the variation curve between the effective modulus and crack cluster density of asphalt concrete containing microcracks and the macrocrack can be obtained, as shown in Figure 2.

**Figure 3:**
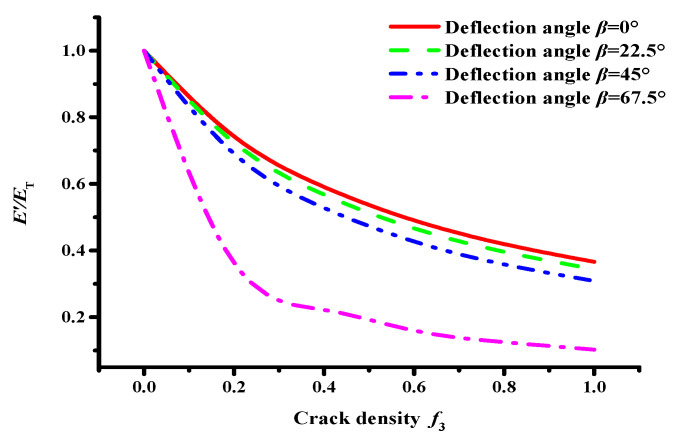
Variation curve of the effective modulus of asphalt concrete with crack density.

The interaction between internal microcracks and the macrocrack in asphalt concrete intensifies as the microcrack density *f*_3_ and deflection angle *β* (see Figure 3) increase. Notably, the interaction effect between the internal microcracks and the main crack is the most significant when the deflection angle *β* is greater than 45°.

To further illustrate the changes in the interaction effect between microcracks and the macrocrack, the Talyor medium method and the DEM were used to establish the meso-structural model of asphalt concrete with a macrocrack and microcracks of different densities. Through an SCB virtual test, the correlation between a multi-crack interaction effect and macrocrack propagation was established in terms of effective modulus change, main crack propagation evolution process, and crack tip stress field change.

## 3. Meso-Structural Model of Asphalt Concrete with Different Crack Distribution Characteristics

### 3.1. Asphalt Concrete Meso-Parameters

According to the research results of the low-temperature laboratory test [42], the gradation of the AC-13 suspended dense structural asphalt concrete is shown in Table 1. And the asphalt adopts PPA-SBS (polyphosphoric acid and styrene butadiene styrene) composite-modified asphalt; its performance grade (PG) is PG 70–28 and the SBS and PPA content is 3% and 1%, respectively. And physical indicators of AC-13 asphalt concrete is shown in Table 2.

**Figure 4:**
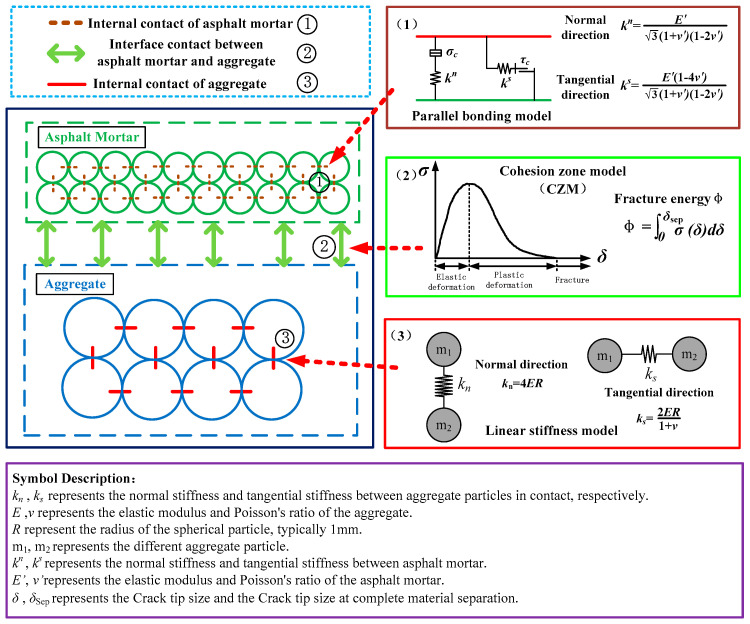
Sketch of interactions and the corresponding constitutive models within the digital sample of asphalt mixtures.

The discrete element meso-structural model of asphalt concrete mainly includes three contact interfaces: the contact between coarse aggregate particles, the contact within the asphalt mortar, and the contact between coarse aggregate particles and asphalt mortar. Therefore, to analyze the crack propagation characteristics of asphalt concrete, the appropriate mechanical model of different contacts [43] should be determined, as shown in Figure 4.

Because aggregates are purely elastic materials, the stiffness model of the contact between aggregates can be described by a spring element, i.e.,

(5) $$\left.\begin{array}{l} k_{n}=4E_{s}R\\ k_{s}=\frac{2E_{s}R}{1+v_{s}} \end{array}\right\}$$

where *k_n_*, *k_s_* denote the normal stiffness and tangential stiffness between aggregate particle contacts; *E_s_*, *ν_s_* denote the dynamic modulus and Poisson’s ratio of the aggregate, respectively. *R* denotes the radius of spherical particles, which is generally taken as 1 mm. As reported in previous studies [44,45], the dynamic modulus, Poisson’s ratio, tensile strength, and coefficient of internal friction of basaltic crushed rock were taken as 55.5 GPa, 0.25, 27.6 MPa, and 0.5, respectively.

Meanwhile, the parallel bond model and the CZM (Cohesive Zone Model) were used to describe the contact behavior between asphalt mortar particles and that between the asphalt mortar and aggregates [46], i.e.,

(6) $$\left.\begin{array}{l} k^{n}=\frac{E_{a}}{\sqrt{3}(1+\nu_{a})\;(1-2\nu_{a})}\\ k^{s}=\frac{E_{a}(1-4\nu_{a})}{\sqrt{3}(1+\nu_{a})(1-2\nu_{a})} \end{array}\right\}$$

where *k^n^, k*^s^ represent the internal normal and tangential stiffness of asphalt mortar; *E_a_*, *ν_a_* represent the dynamic modulus and Poisson’s ratio of asphalt mortar, respectively.

For the contact characteristics of asphalt mortar and aggregate particles, the magnitude of the contact force *σ* between the particles was calculated by tracking the particle position, rotation angle, contact force between particles, and stress measurement circle, i.e.,

(7) $$\sigma =\sqrt{{\left(\sigma_{c}^{n}\right)}^{2}+(\tau_{c}^{n}{)}^{2}}$$

where $\sigma_{c}^{n}$ and $\tau_{c}^{n}$ represent the components of the contact force between particles in the normal and tangential directions, respectively.

The maximum contact force *σ*_max_ between particles can be obtained from the normal force *σ_c_*, tangential force *τ_c_*, and the angle *α* between the direction of contact force and the line through the particle center, i.e.,

(8) $$\sigma_{\max}=(1-\frac{2\alpha }{\pi })\times \sigma_{c}+\frac{2\alpha }{\pi }\times \tau_{c}$$

When the inter-particle contact force *σ* exceeds the maximum contact force *σ*_max_, the inter-particle contact begins to yield or weaken, which indicates a decay in the inter-particle contact force.

**Figure 5:**
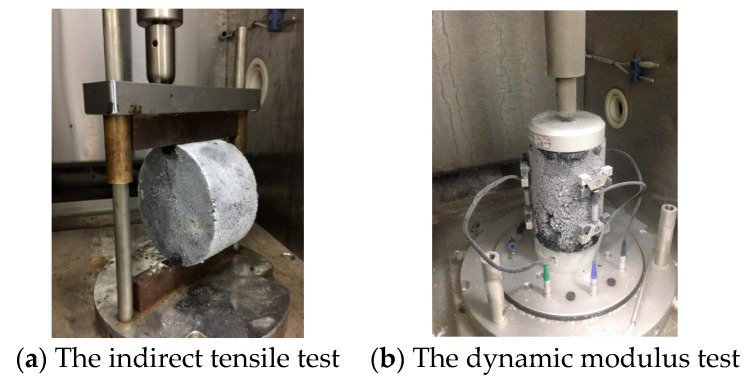
The mechanical properties tests.

The indirect tensile test and the dynamic modulus test were carried out to obtain the mechanical properties of AC-13 asphalt concrete, as shown in Figure 5, Table 3 and Table 4. According to Equations (5)–(8), the meso-mechanical parameters of each material phase at a low temperature of −20 °C are shown in Table 5.

### 3.2. Construction of Microcracks and the Macrocrack

On the PFC2D 5.0 software, using the DEM and the Talyor medium method, a semicircular meso-structural model of asphalt concrete with a radius of 75 mm was established. The macrocrack and microcracks with a uniform randomly distributed location and orientation were constructed using the dfn template and dfn generate commands. Abrupt changes occur in the interaction effect between microcracks and the macrocrack when the macrocrack deflection angle *β* ≥ 45° (see Figure 2). Hence, *β* = 45° and crack length *r* = 2 mm were considered. Meanwhile, the crack sizes of microcracks were all considered to be 1 mm based on the Talyor medium method, and the crack densities *f*_3_ were taken as 0.0, 0.2, 0.4, 0.6, 0.8, and 1.0, respectively.

**Figure 6:**
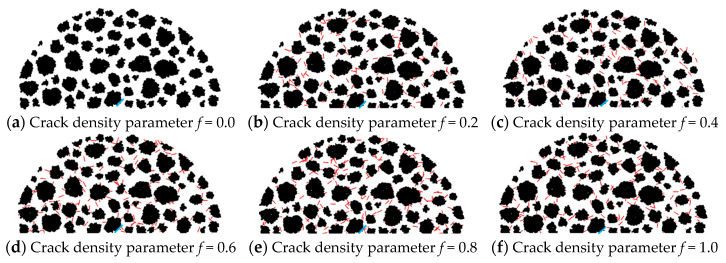
Meso-structural model of asphalt concrete containing microcracks and a macrocrack. (Note: The black particles represent the internal aggregate of asphalt concrete, the red lines represent microcrack clusters, and the blue lines represent the macrocrack).

Figure 6 shows the meso-structural model of asphalt concrete that contains a macrocrack and microcracks of different crack densities. The black particles represent the internal aggregate of asphalt concrete, the red lines represent microcrack clusters, and the blue lines represent the macrocrack (see Figure 6).

### 3.3. SCB Virtual Test

**Figure 7:**
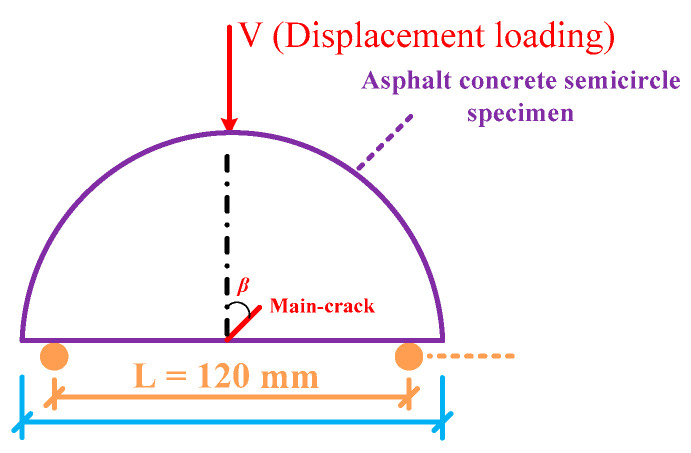
Schematic diagram of the SCB virtual test.

The SCB virtual test of asphalt concrete containing microcracks and a macrocrack (see Figure 7) was performed using the discrete element analysis platform. Two rigid spherical particles were used as pivot points with a spacing *L* of 120 mm; the load was applied by a constant displacement loading of 1 mm/min.

## 4. DEM Results Analysis

**Figure 8:**
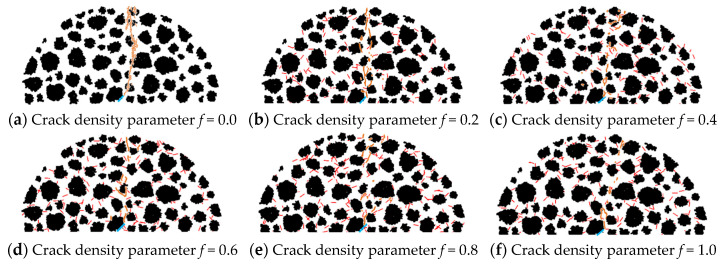
Results of the effect of microcracks on a macrocrack. (Note: The black particles represent the internal aggregate of asphalt concrete, the red lines represent microcrack clusters, the blue lines represent the macrocrack, and the orange lines represent the cracks after propagation).

The SCB virtual test was conducted for asphalt concrete containing macrocracks with different microcrack densities *f_3_* by the discrete element analysis platform (PFC2D). The results are shown in Figure 8, where the solid orange line indicates the crack extension in the material.

It is obvious that there is a large difference in the spatial distribution of the internal crack propagation (see Figure 8), which indicates that the interaction effect between microcracks and the macrocrack changes as the density of microcracks changes.

**Figure 9:**
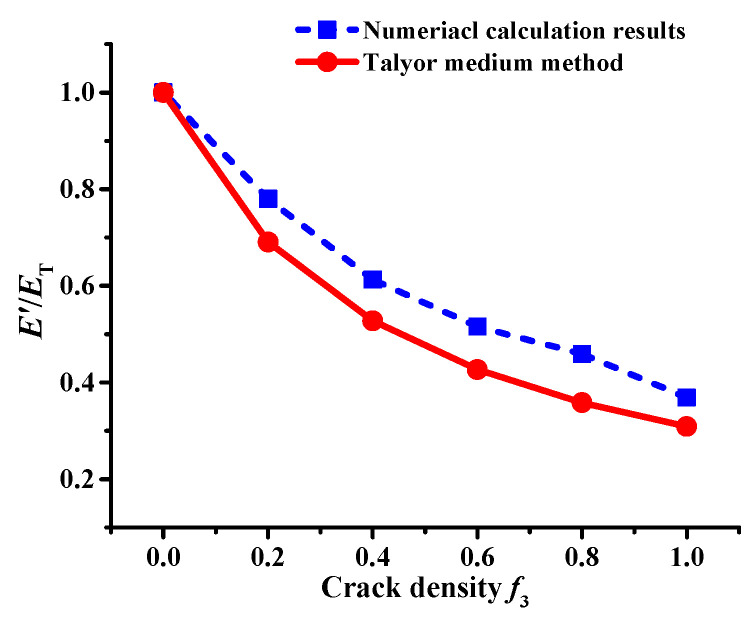
Variation curve of effective modulus of asphalt concrete.

Moreover, the numerical simulation results show the variation curve of the effective modulus of asphalt concrete with a microcrack density of *f*_3_, as shown in Figure 9. It can be seen in Figure 9 that the effective modulus of asphalt concrete with a macrocrack has a nonlinear decreasing trend as the microcrack density *f*_3_ increases. A good correlation exists between the numerical calculation results and the theoretical calculation results of the Talyor medium method, which indicates that the numerical calculation is accurate.

To further analyze the influence of the interaction effect between the microcracks and macrocrack cracks on the macrocrack propagation, the stress–strain relationship, crack extension process, and crack tip stress field are analyzed in the following subsections.

### 4.1. Stress–Strain Curve

**Figure 10:**
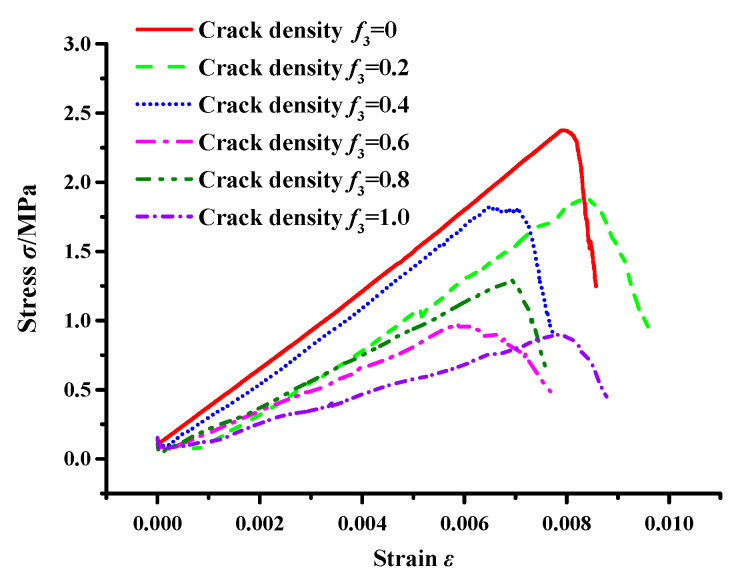
Stress–strain curves of asphalt concrete with main cracks under different microcrack population densities.

The stress–strain variation was monitored by stress measurement circles, and the result is illustrated in Figure 10.

Figure 10 shows that the interaction between microcracks and a macrocrack influences crack propagation until fracture failure occurs in the asphalt concrete, and the interaction effect is substantial. When the crack density *f*_3_ ≤ 0.4, no obvious changes are observed in the fracture stress of the asphalt concrete, meaning that the interaction effect is weak, i.e., the interaction has no obvious effect on the macrocrack propagation (see Figure 8). Moreover, the result also indicates that the interaction at this crack density mainly reduces the effective modulus of the asphalt concrete (see Figure 9).

On the other hand, the interaction effect is enhanced when the crack density *f*_3_ ≥ 0.6, resulting in changes in the crack propagation (see Figure 8); meanwhile, the stress during the damage of asphalt concrete increases and then decreases, that is, the interaction has a shielding effect and an acceleration effect on the internal crack propagation of the material. The shielding and acceleration effects are clearly observed from the parameters related to the crack propagation.

### 4.2. Crack Propagation Process

On the discrete element analysis platform (PFC2D), the crack propagation monitoring procedure was used to determine the crack behavior during the asphalt concrete fracturing in real time. The number of cracks was characterized by the number of particle contact failures in the material; this means that the more particle contact failures, the more the material cracks, indicating that greater energy is required to destroy the asphalt concrete, i.e., the cracking strength of the asphalt concrete is greater.

Therefore, the logarithm and increment of the time step represent the time elapsed at the crack incubation stage, expansion stage, and fracture stage, respectively [47], as shown in Table 6.

Table 6 shows that the increase in the crack density results in a gradual decrease in the crack incubation time, meaning the interaction effect shortens the crack incubation time and speeds up the crack nucleation. Moreover, the table also shows that at the crack extension stage, no significant difference exists in the crack propagation and failure process times when the crack density *f*_3_ ≤ 0.4, further indicating that the interaction effect is weak.

Meanwhile, when the crack density *f*_3_ ≥ 0.6, the time elapsed at the crack extension stage decreases with the increase in crack density; the time elapsed at the fracture stage increases and then decreases (see Table 6). Moreover, the longest elapsed time at the crack extension stage was at the crack density *f*_3_ = 0.6, indicating that the interaction delays the crack extension process; when the crack density *f*_3_ = 0.8, the time elapsed at the fracture stage reached a maximum, meaning the interaction prevents the formation of macroscopic cracks. This result indicates that under these crack densities, the interaction has a shielding effect. Otherwise, at the crack density *f*_3_ = 1.0, a significant decrease occurs in the time elapsed at the crack extension and fracture stages, indicating that the interaction accelerates the crack propagation to macroscopic crack formation, i.e., it has an acceleration effect.

To reveal the generation mechanism of the interaction between microcracks and macrocracks, we analyze the changes in the stress field on the crack tip domain in the following subsection.

### 4.3. Stress Field Analysis for Crack Tip Domain of Main Crack

**Figure 11:**
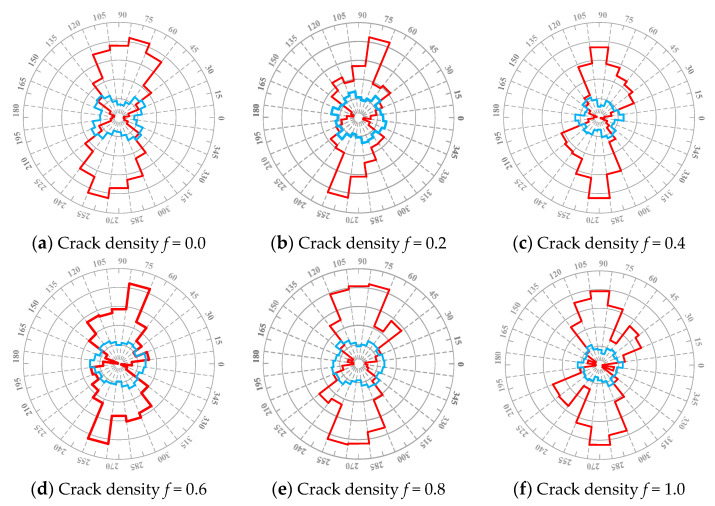
Rose diagram of the stress field in the vertical crack tip domain of the macrocrack in asphalt concrete. (Note: the red line represents the average tensile stress field, and the blue line represents the average shear stress field).

**Figure 12:**
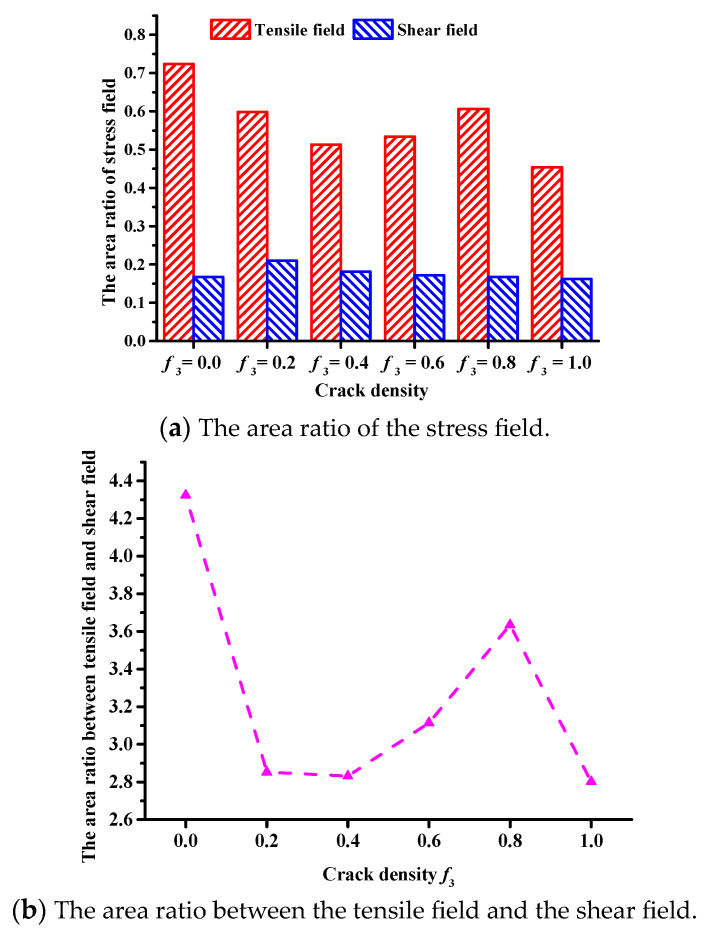
Stress field area ratio of asphalt concrete crack tip.

The subroutine was designed using FISH to identify the stress field of asphalt concrete. The stress field force chain diagrams were obtained, as shown in Figure 11: the red line represents the average tensile stress field and the blue line represents the average shear stress field. The area ratio of the stress field was extracted, as shown in Figure 12.

Figure 11 and Figure 12 show significant changes in the tensile field, unlike the shear field, which only changes slightly. This result indicates that as the crack density increases, the interaction causes the asphalt concrete failure to change from tensile stress failure to shear stress failure. However, when the crack density *f*_3_ = 0.6 and 0.8, the increments of the tensile field and shear field (see Figure 12b) indicate that the crack shielding effect predominantly causes tensile stress damage to the asphalt concrete, delaying the macroscopic crack formation (see Table 6) and increasing the low-temperature crack resistance of the asphalt concrete (see Figure 10).

Meanwhile, Figure 11 and Figure 12 also show that the area ratio of the tensile field and the area ratio between the tensile and shear fields decreases considerably when the crack density *f*_3_ = 1.0. This illustrates that the interaction predominantly induces shear stress damage to the asphalt concrete, which accelerates the crack propagation to a macroscopic crack formation (see Table 6).

## 5. Conclusions

A meso-structural model of asphalt concrete with a macrocrack and microcracks of different crack densities was established using the Talyor medium method and DEM. An SCB virtual test was conducted to analyze the influence of the interaction between microcracks and macrocracks on crack propagation. The analysis considered factors such as effective modulus, stress–strain curve, crack propagation process, and stress field. The conclusions are as follows:

(1) A good correlation exists between the Talyor medium model and the numerical results. The interaction effect persists throughout the entire crack propagation process, and it is weak when the crack density is low, which only reduces the effective modulus of the asphalt concrete; when the crack density is greater than 0.4 (*f*_3_ > 0.4), the interaction has crack shielding and acceleration effects, respectively.
(2) Regarding the crack extension process, when the crack density *f*_3_ = 0.6 and 0.8, the interaction delays the time elapsed at the crack extension stage and fracture stage, i.e., it has a crack shielding effect; when the crack density *f*_3_ = 1.0, the interaction significantly reduces the time elapsed at the crack extension stage and fracture stage, i.e., it has a crack acceleration effect.
(3) According to the results of the stress field, the interaction mainly influences the tensile field, and no obvious changes occur in the shear field. When the crack density *f*_3_ = 0.8, the area ratio between the tensile and shear fields is increased, meaning the interaction has a crack shielding effect, causing the asphalt concrete to mainly undergo tensile stress damage; meanwhile, the area ratio between the tensile and shear fields is decreased as the crack density increases, indicating that the interaction has a crack acceleration effect, which transforms the tensile stress damage to shear stress damage.

## 6. Research Significance and Perspectives

The microcrack randomness in the spatial distribution results in changes in the macrocrack tip stress field, leading to changes in macrocrack propagation behavior. This study deals with crack propagation and the interaction between micro- and macrocracks by combining the Talyor medium method and the DEM. The results provide a predictive theoretical and numerical model for low-temperature cracking of asphalt pavement, and theoretical support for the design, maintenance, and upkeep of long-life pavement.

Moreover, by combining the Digital Image Correlation (DIC) and the Digital Speckle Method (DSM), the toughening effect of aggregate spatial and microcrack groups can be revealed by further analyzing the full-field strain results and the numerical simulation results. Moreover, this approach would lay the foundation for further revealing the cracking behavior of asphalt pavement affected by different factors such as temperature and asphalt binders.

## Figures and Tables

**Table 1:** Gradation of AC-13 asphalt concrete.

Mess size (mm)	16.0	13.2	9.5	4.75	2.36	1.18	0.60	0.30	0.15	0.075
Pass rate (%)	100.0	97.5	84.0	62.5	42.5	32.0	24.0	15.5	11.0	6.0

**Table 2:** Physical indicators of AC-13 asphalt concrete.

Oil–Stone Ratio (%)	Gross Volumetric Density (g/cm^3^)	Marshall Stability (kN)	Porosity (%)	Flow Value (mm)	Void Filled with Asphalt VFA (%)	Maximum Theoretical Density (g/cm^3^)
6.27	2.547	12.48	2.1	4.34	76.2	2.610

**Table 3:** The indirect test result of AC-13 asphalt concrete at −20 °C.

Items	Values
Failure load (kN)	35.91
Splitting strength (MPa)	3.553
Failure tensile strain (*μ*_ε_)	0.0103
Destructive stiffness modules (MPa)	596.85

**Table 4:** The dynamic modulus test result of AC-13 asphalt concrete at −20 °C.

Temperature (°C)	Dynamic Modulus (MPa)					
	0.1 Hz	0.5 Hz	1 Hz	5 Hz	10 Hz	25 Hz
−20	20,973	23,835	24,933	26,991	27,717	28,486

**Table 5:** Calculation parameters of AC-13 asphalt concrete with a parallel bond model at −20 °C.

Material Phase Class	Calculated Parameters	
Coarse aggregate	Dynamic modulus (GPa)	55.5
	Tensile strength *σ* (MPa)	27.6
	Poisson’s ratio *ν_s_*	0.23
	Normal stiffness *k_n_* (MPa)	222
	Tangential stiffness *k_s_* (MPa)	90.24
Asphalt concrete	Void ratio (%)	2
	Particle density (kg/m^3^)	2582
Asphalt mortar	Inter-particle contact modulus *E_c_* (GPa)	0.832
	Particle normal to tangential stiffness ratio *k^n^/k^s^*	1
	Parallel bond modulus *E_c_*^′^ (GPa)	0.596
	Parallel bond normal to tangential stiffness ratio *k^n^_c_/k^s^_c_*	0.667/0.133
	Inter-particle friction coefficient *f_s_*	0.5
	Average normal strength of parallel bond *σ_c_* (MPa)	3.553
	Standard deviation of parallel bond normal strength (MPa)	1
	Average tangential strength of parallel bond *τ_c_* (MPa)	3.553
	Standard deviation of parallel bond tangential strength (MPa)	55.5

**Table 6:** Relevant parameters during crack propagation.

Crack Density Parameter *f*_3_	Crack Incubation Stage		Crack Extension Stage		Fracture Stage	
	Number of Cracks	Time Step Log *lgt*	Number of Cracks	∆_1_	Number of Cracks	∆_2_
0	1	4.39	33	0.22	446	0.02
0.2	1	4.20	29	0.27	242	0.05
0.4	1	4.04	28	0.24	218	0.06
0.6	1	4.01	14	0.7	144	0.04
0.8	1	3.81	15	0.42	164	0.06
1	1	3.65	12	0.32	136	0.04

Note: ∆_1_, ∆_2_ represent the time elapsed at the crack extension stage and fracture stage, respectively.

## Data Availability

The original contributions presented in the study are included in the article, further inquiries can be directed to the corresponding author.
